# Association between interleukin-27 and suicidal ideation: a Mendelian randomization analysis and peripheral blood experimental study

**DOI:** 10.3389/fpsyt.2025.1678780

**Published:** 2026-01-05

**Authors:** Xiwen Hu, Xiaowen Yin, Shengdong Wang, Chunyan Zhu, Xuan Ju, Zhonglin Tan, Lingna Lu

**Affiliations:** 1 Department of Psychiatry, Affiliated Mental Health Center and Hangzhou Seventh People’s Hospital, Zhejiang University School of Medicine, Hangzhou, Zhejiang, China; 2 Zhejiang Academy of Traditional Chinese Medicine, Department of Clinical Laboratory, Tongde Hospital of Zhejiang Province, Hangzhou, Zhejiang, China

**Keywords:** depression, suicide, cytokines, suicidal ideation, Mendelian randomization, IL-27

## Abstract

**Background:**

Suicide is a leading global cause of mortality, with major depressive disorder (MDD) contributing significantly. Neuroimmune mechanisms, particularly inflammation, are increasingly recognized in the pathophysiology of depression and suicidal ideation. This study investigated the relationship between inflammatory cytokines and suicidal ideation in patients with MDD.

**Methods:**

A two sample Mendelian randomization analysis using Genome-Wide Association Study data was performed to evaluate the associations between 16 inflammatory cytokines and suicidal ideation. Then the patients with MDD, stratified by suicidal ideation severity were assessed for peripheral cytokine levels (interleukin [IL])-2, IL-4, IL-6, IL-10, interferon-gamma [IFN-γ], IL-17, IL-12, IL-27, and tumor necrosis factor-α) using flow cytometry and enzyme-linked immunosorbent assay.

**Results:**

MR analysis revealed significant associations of tumor necrosis factor-related apoptosis-inducing ligand (TRAIL) and IL-27 with negative and positive effects, respectively. Individuals with high suicide risk exhibited elevated IL-27, IL-12, IFN-γ and IL-4 compared with low suicide risk.

**Conclusion:**

There are genetic associations between IL-27 and suicidal ideation, which is biologically corroborated by elevated peripheral IL-27 levels in high-risk suicidal individuals, highlighting its potential as a clinically viable biomarker for assessing suicidal risk in depressive patients.

## 1 Introduction

Globally, suicide is a major public health concern, ranking among the leading causes of mortality and representing the second most prevalent contributor to preventable deaths across populations (1). The Diagnostic and Statistical Manual of Mental Disorders, Fifth Edition, designates suicidal behavior as a discrete diagnostic entity. However, population-based studies indicate that nearly three-fifths of completed suicides exhibit clinically significant comorbidity with major depressive disorder (MDD) (2).

MDD is a prevalent mental health disorder characterized by persistent low mood, diminished interest, and sleep disturbances, with known risk factors including a family history of depression, childhood trauma, and life stressors (3). Given the substantial morbidity and mortality rates associated with MDD, a comprehensive understanding of its pathophysiological mechanisms is essential for developing effective preventive and therapeutic interventions.

Recent studies have highlighted neuroimmune mechanisms as pivotal components in the pathophysiology of depression (4). Research has shown that the concentrations of inflammatory biomarkers—including soluble interleukin-2 receptor (sIL-2R), tumor necrosis factor-alpha (TNF-α), interleukin (IL)-6, IL-12, and IL-18—are significantly higher in the bloodstream of individuals with depression than in those without, underscoring the critical role of inflammatory processes in this condition (5).

Emerging evidence supports inflammatory pathways as a plausible biological link between depressive states and suicidal manifestations. Elevated levels of immune-related biomarkers, including IL-6, TNF-α, and C-reactive protein, have been consistently observed in individuals with clinical depression or suicidal behaviors, suggesting that immune dysregulation may underlie the pathophysiology of MDD and suicide risk (6, 7).

Anti-inflammatory interventions have shown promise in alleviating depressive symptoms in some patient subgroups; however, inconsistent findings underscore the complexity of neuroimmune involvement (8). Clarifying these pathways may improve our understanding of MDD and identify inflammation as a target for mitigating suicidal tendencies in clinical practice.

Despite these insights, the precise mechanisms linking inflammation and suicide in MDD remain poorly understood. Some meta-analyses indicate that elevated IFN-γ and IL-6 levels are associated with increased suicide risk (9, 10). However, other studies report diminished or stable levels of pro-inflammatory cytokines in suicidal individuals compared to non-suicidal controls (11, 12). These inconsistencies may result from differences in study populations, assessment methods, and analytical approaches, highlighting the need for a more rigorous evaluation of cytokine–suicidality associations. In this study, we aimed to address this knowledge gap by integrating real-world clinical data with Mendelian randomization (MR) analysis to investigate the role of inflammatory cytokines in suicidality among patients with MDD.

This study aims to provide novel insights into the intricate interplay between inflammation and suicidality, offering potential targets for early intervention and improved clinical management.

## 2 Subjects and methods

### 2.1 MR analysis

#### 2.1.1 Study design

This study employed a two-sample MR design to investigate the potential relationship between circulating inflammatory cytokines and suicidal ideation. The MR method relies on genetic variants, specifically single nucleotide polymorphisms (SNPs), as instrumental variables (IVs) to estimate the relationships. The MR method requires instrumental variables to satisfy the following three key assumptions:

1. Relevance Assumption: Genetic variants must be significantly associated with cytokine levels.
2. Independence Assumption: Genetic variants should not be associated with any potential confounding factors that influence both the exposure and the outcome.
3. Exclusivity Assumption: Genetic variants must affect the outcome solely through their association with the exposure.

#### 2.1.2 Data sources

Genome-wide association study (GWAS) summary statistics for cytokines were derived from the IEU OpenGWAS (MRC-IEU) database, which provides publicly available GWAS data for multiple cytokines. The cytokines selected for analysis in this study included IL-2, IL-4, IL-5, IL-9, IL-13, IL-17, IL-27, TNF-α, TNF-β, IFN-γ, granulocyte-colony stimulating factor, macrophage colony stimulating factor, transforming growth factor-beta, IL-12p70, TNF-related apoptosis-inducing ligand (TRAIL), and IL-6. These cytokine datasets each have independent GWAS IDs and sample sizes clearly reported in the database. The IEU OpenGWAS (ukb-e-20513_CSA) database covers UK Biobank Field 20513 (“recent thoughts of suicide or self-harm,” Patient Health Questionnaire-9 [PHQ-9] item 9) and includes 1,165 cases. The released summary statistics combine suicidal ideation and non-suicidal self-harm ideation and are not MDD-restricted.

#### 2.1.3 Selection of instrumental variables

Instruments were selected using LD clumping (r²<0.001, 10,000 kb, EUR). The instrument strength was adequate (per-SNP F; all F>10; IL-27: median 26.2, IQR 21.8–28.3, min 21.5; TRAIL: median 22.9, IQR 21.6–66.1, min 20.9), and directionality was supported by Steiger testing (IL-27: 11/11 passed; TRAIL: 19/19 passed). The primary MR analysis was performed using the inverse-variance weighted (IVW) method. This method provides robust estimates of correlations under the assumption of valid IVs without pleiotropy. To support the independence assumption, we screened all instruments in large external GWASs of body mass index, smoking, alcohol consumption, education/socioeconomic status, neuroticism, C-reactive protein (CRP), lipids, Type 2 diabetes, and blood pressure and repeated IVW after excluding SNPs with genome-wide significant associations (p<5×10^−8^). Additionally, complementary MR analyses including MR-Egger regression, weighted median, simple mode, and weighted mode methods were employed for sensitivity checks and validation of IVW results.

#### 2.1.4 Statistical methods

To verify the robustness of the MR findings, comprehensive sensitivity analyses were conducted: Cochran’s Q-test was used to evaluate heterogeneity among SNPs where significant heterogeneity (P < 0.05) necessitated the use of random-effects IVW; otherwise, fixed-effects IVW was considered valid. In horizontal pleiotropy test, MR-Egger intercept analysis and MR-PRESSO global test were used to detect directional pleiotropy (P > 0.05 indicated no significant pleiotropy). In Leave-One-Out analysis, each SNP was sequentially removed to evaluate if single SNPs disproportionately influenced the results, confirming robustness. All MR analyses were conducted in R software (version 4.4.2) utilizing the TwoSampleMR and MR-PRESSO packages.

### 2.2 Subjects

This study enrolled 62 patients (14 males and 48 females; mean age: 25 ± 7 years) treated at the Department of Psychiatry of Hangzhou Seventh People’s Hospital between April 2022 and April 2023. Peripheral venous blood samples (1 mL) were collected from all patients before hospitalization.

The inclusion criteria were 1) a current diagnosis of MDD confirmed by Mini-International Neuropsychiatric Interview (M.I.N.I.). Exclusion criteria included: 1) signs of active infection; 2) autoimmune diseases or other severe somatic illnesses; 3) comorbidity with schizophrenia, intellectual disability, or other major psychiatric disorders; and 4) pregnancy.

Patients were categorized into two groups based on the suicidal ideation section of the M.I.N.I., as assessed by professional psychiatrists: low suicidal ideation (LSI) and high suicidal ideation (HSI), comprising 38 and 24 participants, respectively.

Twenty-eight healthy individuals without a history of mental disorders were recruited as controls and participated voluntarily. Peripheral venous blood samples (1 mL) were also collected from all healthy controls using the same procedure. Exclusion criteria for controls were the same as those applied to the patient group. The control group included 9 males and 19 females, with a mean age of 26 ± 7 years.

This study was approved by the Ethics Committee of Hangzhou Seventh People’s Hospital (Approval No. 2022-026) and the Ethics Committee of Tongde Hospital of Zhejiang Province (Approval No. 2022-YAN-NO.019-JY). All participants provided informed consent.

### 2.3 Data collection

#### 2.3.1 Measurement of IL-2, IL-6, IL-10, interferon-gamma (IFN-γ), IL-17, IL-4, IL-12, and TNF-α levels using flow cytometry

Peripheral venous blood samples (1 mL) were collected from all participants (patients and healthy controls) and deposited in tubes containing ethylenediaminetetraacetic acid dipotassium salt anticoagulant. Samples were centrifuged at 1000 ×g for 30 min to isolate plasma. The plasma concentrations of IL-2, IL-6, IL-10, IFN-γ, IL-17, IL-4, IL-12, and TNF-α were measured using a flow cytometry microsphere array technique.

A standard curve was established via serial dilution of calibration standards to generate eight concentration gradients (10,000; 2,500; 625; 156.3; 39.1; 9.8; 2.4; and 0 pg/mL). For immunoassay implementation, 25 µL aliquots of calibration standards and plasma samples were added to reaction tubes, followed by 25 µL each of capture microsphere antibodies, detection antibodies, and assay buffer to form the immunocomplex matrix. The reaction mixture was incubated in a light-protected, agitated environment at 25 ± 2°C for 120 mins to facilitate antigen-antibody binding.

After the primary incubation, 25 µL streptavidin-phycoerythrin conjugate was added to each tube and incubated for 30 min under the same conditions to amplify the fluorescent signal. Post-incubation, samples were centrifuged at 250 × g for 5 min, and the supernatant was aspirated. Cytokine concentrations (IL-2, IL-4, IL-6, IL-10, IFN-γ, IL-17, IL-12, and TNF-α) were quantified by analyzing microsphere complexes resuspended in 200 µL phosphate-buffered saline (pH 7.4) using a Navios flow cytometer (Beckman Coulter, USA) with four-parameter logistic regression analysis based on the standard curve.

This protocol demonstrated excellent reproducibility with coefficients of variation (CV) < 5% across all cytokine assays. All procedures followed the manufacturer’s instructions for the multiplex cytokine detection kit (Qingdao Reeskail Bio-Tech Co., Ltd. Qingdao, China).

#### 2.3.2 Measurement of IL-27 levels using ELISA

Peripheral venous blood samples (1 mL) were collected from all participants (patients and healthy controls) and deposited in tubes containing ethylenediaminetetraacetic acid dipotassium salt anticoagulant. Samples were centrifuged at 1000 × g for 30 min to isolate plasma. A standard curve was established via serial dilution of calibration standards to generate eight concentration gradients (1,000; 500;250; 125; 62.5; and 0 pg/mL).

Blank and sample wells were set up on the enzyme-labeled microplate, with the addition of a 40-μL sample and 10 μL of biotinylated anti-IL-27 antibody to each sample well, followed by 50 μL of an enzyme-labeled reagent (excluding blank wells). The plate was sealed and incubated at 37°C for 30 min, and then washed five times with wash buffer. Then, 50 μL Chromogen A and 50 μL Chromogen B were added to each well and incubated at 37°C in the dark for 10 min, and the reaction was stopped with 50 μL stop solution. OD450 nm was measured, a standard curve was generated by four-parameter fitting, and sample concentrations were calculated using the curve equation.

All procedures followed the manufacturer’s instructions for the multiplex cytokine detection kit (WuHan Bioswamp Bio-Tech Co., Ltd. Wuhan, China).

### 2.4 Statistical methods

#### 2.4.1 Software and tools

Statistical analyses were performed using the Statistical Package for Social Sciences version 18.0 (IBM, Armonk, NY, USA) and Graphpad Prism 8 (GraphPad Software, San Diego, CA, USA). MR analyses were conducted in R (version 4.4.2) (Posit Software, Boston, MA, USA) using the TwoSampleMR package. MR results, including scatter, leave-one-out (LOO) sensitivity, and forest plots, were analyzed using the ggplot2 package.

#### 2.4.2 Continuous variable analysis

The normality of the dataset was assessed using the Shapiro–Wilk test. Results were expressed as mean ± standard deviation ( $\bar{\text{X}}$ ± s) for normally distributed data, and between-group comparisons were performed using independent t-tests. For non-normally distributed data, results were expressed as medians (P25, P75), and comparisons were made using the Mann–Whitney U test (for two groups). With regard to binary variables, the chi-square test was used to compare the sex distribution between the patient and control groups.

## 3 Results

### 3.1 MR analysis

**Figure 1:**
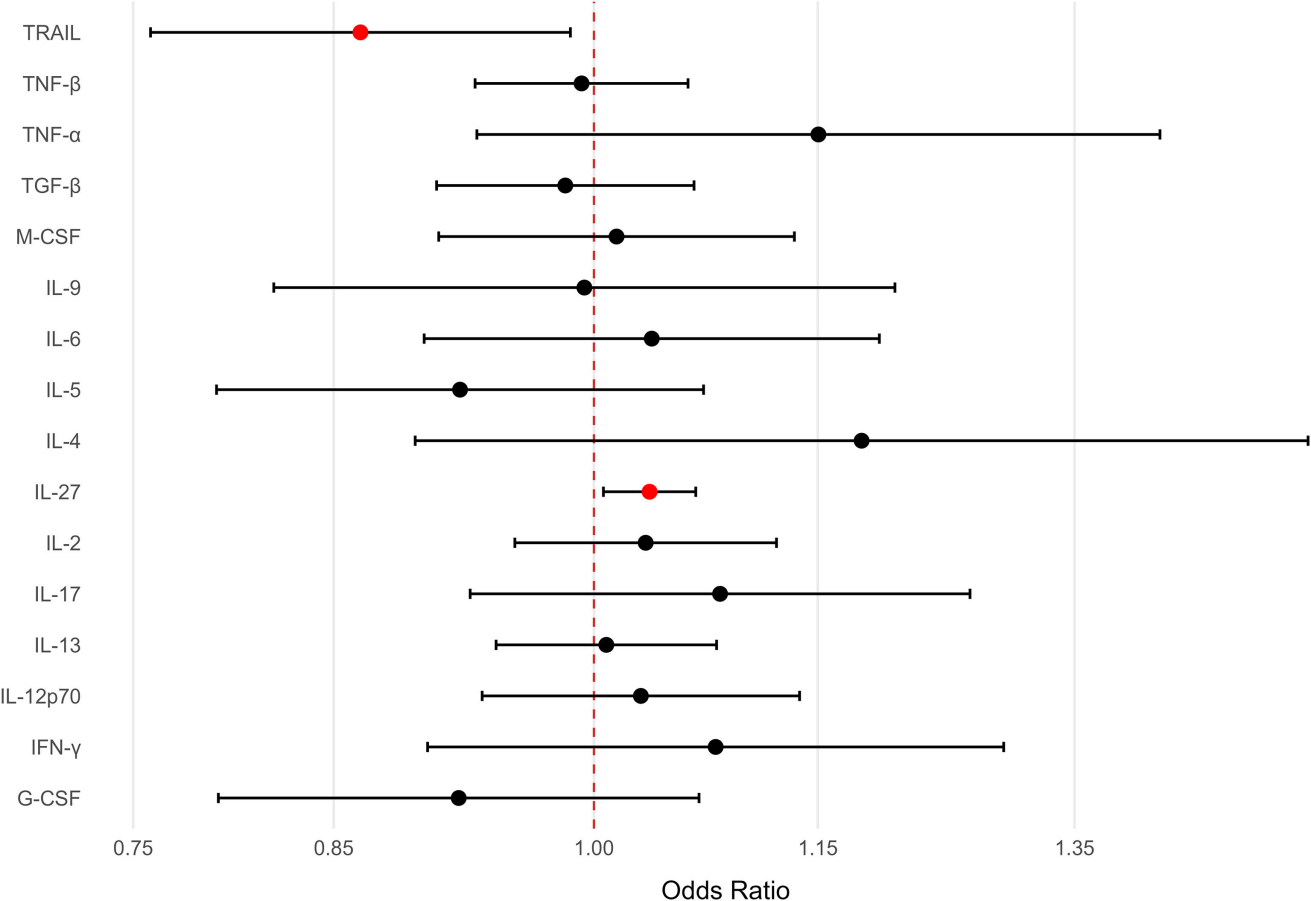
Forest plot of Mendelian randomization estimates for cytokines versus recent thoughts of suicide or self-harm (UKB Field 20513). IVW Mendelian randomization estimates as odds ratios (ORs; per 1 standard deviation increase in genetically proxied cytokines). The points show ORs with 95% confidence intervals (CIs); the vertical dashed line denotes OR = 1 (no association). The red points indicate p<0.05 (uncorrected), and the black/white points are not significant after this threshold. Cytokines are ordered for readability.

A two-sample MR analysis was performed using publicly available GWAS summary statistics to investigate the potential correlations of inflammatory cytokines (IL-2, IL-4, IL-5, IL-9, IL-13, IL-17, IL-27, TNF-α, TNF-β, IFN-γ, granulocyte-colony stimulating factor, macrophage colony stimulating factor, transforming growth factor-β, IL-12p70, TNF-related apoptosis-inducing ligand (TRAIL), and IL-6) with suicidal ideation (**Figure 1**).

**Figure 2:**
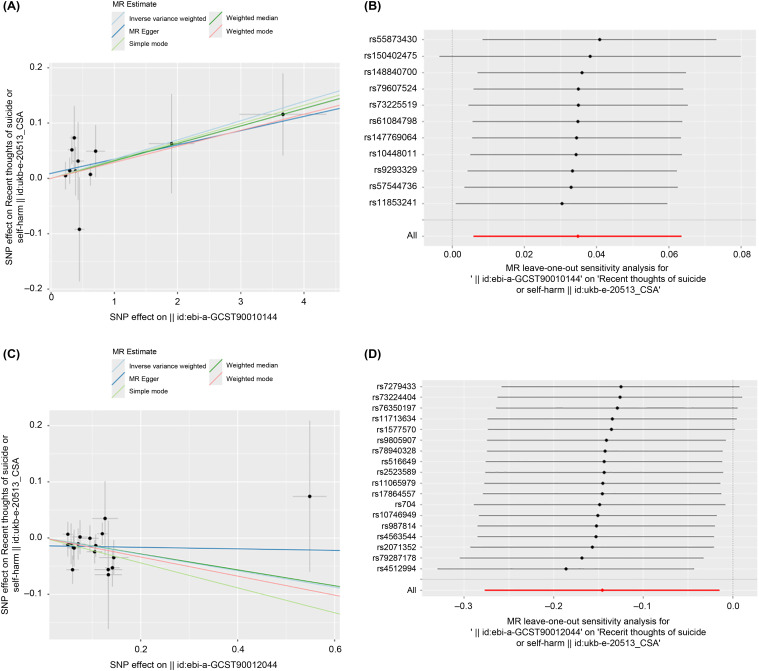
Mendelian Randomization (MR) analysis of TRAIL and IL-27 on suicidal ideation. **(A, C)** Scatter plots of SNP effect estimates for exposure (TRAIL or IL-27) versus outcome (suicidal ideation). Each point represents a single nucleotide polymorphism (SNP), and error bars indicate standard errors. The solid line represents the Inverse Variance Weighted (IVW) estimate, indicating the correlation of IL-27 **(A)** and TRAIL **(C)** with suicidal ideation. **(B, D)** Leave-One-Out (LOO) sensitivity analysis for IL-27 **(B)** and TRAIL **(D)**. Each row represents the effect estimate when excluding one SNP at a time, with error bars representing 95% confidence intervals. The red bar at the bottom represents the overall MR estimate, which remains statistically significant even when any single SNP is removed, suggesting that the correlation is robust and not driven by a single instrumental variable.

Only two cytokine datasets—TRAIL (ebi-a-GCST90012044) and IL-27 (ebi-a-GCST90010144)—demonstrated significant correlations with suicidal ideation based on IVW analysis. A significant association between IL-27 (ebi-a-GCST90010144) and suicidal ideation was observed (odds ratio [OR] = 1.04, 95% confidence interval [CI]: 1.01–1.07, P = 0.018), indicating that higher IL-27 levels may increase the risk of suicidal ideation (**Figure 2A**). No instrument was externally flagged; therefore, exclusion results were identical. A significant association between TRAIL (ebi-a-GCST90012044) and suicidal ideation was observed (OR = 0.86, 95% CI: 0.76–0.98, P = 0.029); this suggested that higher TRAIL levels may have a protective effect (**Figure 2C**). Following removal of five external-hit instruments, the association persisted with similar magnitude (OR = 0.84, 95% CI: 0.72–0.98, P = 0.025).

Heterogeneity analysis: Cochran’s Q-test showed no significant heterogeneity for IL-27 (IVW: Q = 5.698, P = 0.840) or TRAIL (IVW: Q = 10.849, P = 0.864), indicating consistent effects across SNPs.

Horizontal pleiotropy analysis: MR-Egger intercepts were non-significant for IL-27 (intercept = 0.0091, P = 0.546) and TRAIL (intercept = -0.0139, P = 0.408), suggesting no evidence of directional pleiotropy.

LOO Sensitivity Analysis: Sequential exclusion of individual SNPs did not materially alter the correlation estimates, further supporting the stability of the associations (**Figures 2B, D**).

### 3.2 Demographic characteristics of the study participants

**Table 1** summarizes the demographic characteristics of the study participants. A total of 62 patients with MDD and 28 healthy controls were included in the final analysis. There were no significant differences in age (P = 0.328) or sex distribution (P = 0.366) between the patient and control groups; thus, the groups were well-matched at baseline.

**Table 1:** Demographic characteristics of the study participants.

Characteristic	Depression group	Control group	Statistic	P-value
	(n = 62)	(n = 28)		
Sex, n (%)			χ² = 0.927	0.366
Male	14 (22.6)	9 (32.1)		
Female	48 (77.4)	19 (67.9)		
Age (years)	23 (29, 26)	24.5 (21, 31)	Z = -0.979	0.328

Age values are presented as median (P25, P75).

### 3.3 Differential analysis of inflammatory biomarkers in patients with MDD

Quantitative profiling demonstrated significantly elevated plasma concentrations of IL-6, IL-10, IFN-γ, IL-17, TNF-α, and IL-27 in patients with MDD compared to healthy controls (P < 0.05). However, no statistically significant differences between the two groups were observed in IL-2, IL-4, and IL-12 expression levels (**Table 2**).

**Table 2:** Differential plasma cytokine profiles between the major depressive disorder and control groups.

Cytokine (pg/mL)	Depression group	Control group	p-value
	(n = 62)	(n = 28)	
IL-2	0.00 (0.0, 8.06)	0.42 (0.24, 1.07)	0.622
IL-6	10.46 (4.49, 20.90)	0.57 (0.23, 1.48)	<0.0001^*^
IL-10	3.19 (1.94, 5.14)	0.30 (0.22, 0.45)	<0.0001^*^
IFN-γ	10.66 (6.40, 17.01)	0.0 (0.00, 3.02)	<0.0001^*^
IL-17	2.98 (1.83, 4.50)	0.0 (0.0, 0.0)	<0.0001^*^
IL-4	0.0 (0.0, 0.12)	0.0 (0.0, 0.0)	0.12
IL-12	0.26 (0.09, 0.76)	0.32 (0.15, 0.70)	0.283
TNF-α	2.59 (1.31, 5.31)	0.19 (0.0, 1.07)	<0.0001^*^
IL-27	373.45 (290.26, 456.01)	220.04 (188.51, 258.08)	<0.0001^*^

Values are presented as median (P25, P75). Comparisons were made using the Mann–Whitney U test.

*Statistically significant at P < 0.05.

IL, Interleukin; IFN-γ, Interferon-gamma; TNF-α, Tumor necrosis factor-alpha.

### 3.4 Differential cytokine profiles across suicidality severity in patients with MDD

**Figure 3:**
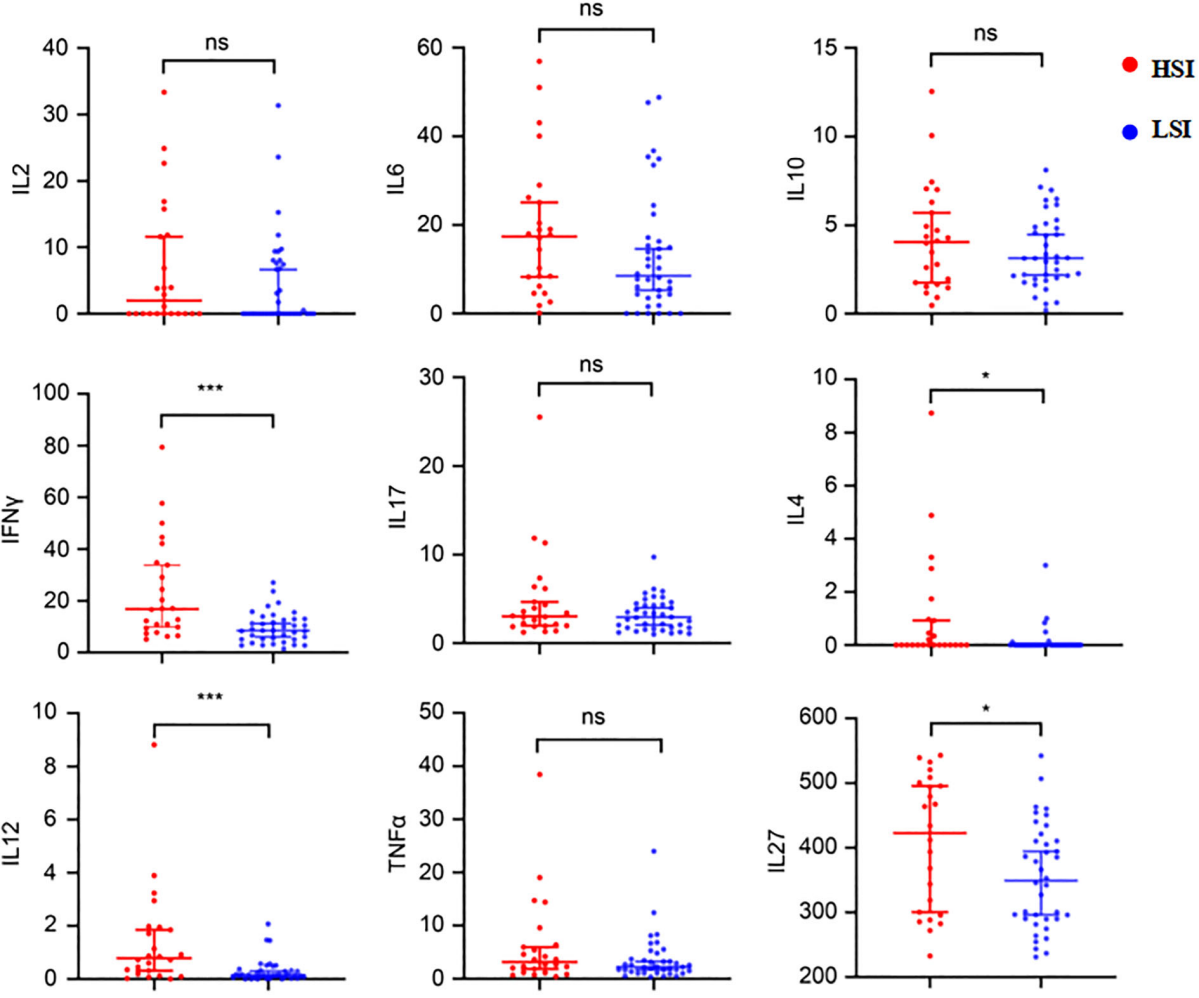
Differential plasma cytokine profiles between LSI and HSI. * Statistically significant at P < 0.05. *** Statistically significant at P < 0.001. ns, not significant (p ≥ 0.05).

Quantitative profiling demonstrated significantly elevated plasma concentrations of IL-4, IL-12, IFN-γ, and IL-27 in patients with HSI compared to LSI (P < 0.05). However, no statistically significant differences between the two groups were observed in IL-2, IL-6, IL-10, IL-27, and TNF-α expression levels (**Table 3**, **Figure 3**).

**Table 3:** Differential plasma cytokine profiles between the low suicidal ideation (LSI) and high suicidal ideation (HSI) groups.

Cytokine (pg/mL)	LSI	HSI	p-value
	(n = 38)	(n = 24)	
IL-2	0.00 (0.0, 7.68)	1.98 (0.00, 11.78)	0.422
IL-6	8.52 (3.78, 16.49)	17.38 (6.71, 25.92)	0.0613
IL-10	3.13 (2.01, 4.95)	4.04 (1.68, 6.15)	0.573
IFN-γ	8.51 (1.71, 4.38)	16.81 (9.53, 34.50)	0.0006^*^
IL-17	2.93 (1.71, 4.38)	3.03 (1.96, 5.79)	0.3194
IL-4	0.0 (0.0, 0.0)	0.0 (0.0, 0.96)	0.0110^*^
IL-12	0.14 (0.07, 0.33)	0.78 (0.22, 1.93)	0.0003^*^
TNF-α	2.21 (1.29, 3.96)	3.13 (1.34, 6.27)	0.2239
IL-27	349.27 (289.86, 413.18)	422.76 (296.78, 499.29)	0.0406^*^

Values are presented as median (P25, P75). Comparisons were made using the Mann–Whitney U test.

IL, Interleukin; IFN-γ, Interferon-gamma; TNF-α, Tumor necrosis factor-alpha. * Statistically significant at P < 0.05.

## 4 Discussion

Activation of immune-inflammatory pathways is a well-established contributor to the onset and progression of depression, with cytokines playing pivotal roles in neuroimmune modulation (4). Historically, the blood-brain barrier (BBB)was believed to grant the central nervous system (CNS) total independence and protection from peripheral immune processes. However, current understanding recognizes that in certain states, its permeability could either influence or be influenced by central neuroinflammation (13). Depressive-like symptoms induced by chronic restraint stress (CRS) in rats are closely associated with the recruitment of peripheral CD4^+^ Th17 cells into the central nervous system, the peripheral immune response is propagated into the CNS through a leaky BBB, thereby progressively exacerbates neuroinfammation and disease symptoms (14). Another study found that chronic unpredictable mild stress (CUMS) treatment induced aberrant activation of microglia and upregulation of circular RNA UBE2K (circ-UBE2K) in mice, thereby exacerbating neuroinflammation and neuronal damage. Downregulation of circ-UBE2K ameliorated microglial activation and depressive-like behaviors while reducing the expression of inflammatory factors (15). Both preclinical and clinical studies have found higher levels of IL-1β, TNF-α, and IL-6 in peripheral blood and brain tissue of patients with depression (16). Our findings revealed significantly elevated circulating levels of IL-6, IFN-γ, IL-17, TNF-α, IL-10, and IL-27 in individuals with depression compared to healthy controls. This coordinated activation of the pro-inflammatory response system and its counter-regulatory mechanisms illustrates a disrupted cytokine network, marked by regulatory disequilibrium between neurotoxic inflammatory signaling and immunomodulatory feedback loops in MDD pathogenesis (17, 18). Recent MR studies have helped clarify the potential correlations of inflammatory cytokines with depression. A 2023 large-scale two-sample MR study analyzing 41 inflammation-related factors across over 130,000 depression cases found that only a few cytokines, such as IL-18, IL-1β, and RANTES, exhibited protective associations with depression risk (19). Collectively, these findings demonstrate that inflammatory factors critically contribute to the symptom presentation, pathogenesis, and clinical progression of depression.

Beyond depression, inflammatory responses have also been linked to suicidal ideation and behavior (20). The overarching narrative that emerges is that suicidality is linked to a specific dysregulation of the neuroimmune axis, characterized less by blanket inflammation and more by a critical imbalance between pro-inflammatory and compensatory immunoregulatory pathways. Compiled evidence demonstrates a coordinated upregulation of key pro-inflammatory cytokines in suicidal states. Moreover, accumulating evidence from numerous reports converges to suggest that IL-6 levels are frequently elevated in individuals diagnosed with either MDD or bipolar disorder (21, 22). Another study of suicide attempts reported significantly higher serum IL-6 levels in individuals who attempted suicide than in the control group, although the *IL6R* polymorphism rs2479409 was not associated with suicide attempts (23). However, a post-mortem study of 234 suicide cases revealed that IL-6 (rs1800795) was significantly associated with death by suicide (24). This central pro-inflammatory driver is complemented by elevated TNF-α levels associated with increased suicidal symptoms in several cohorts (21, 25). Furthermore, IL-1β has been implicated in suicide risk, with associations observed at both the genetic level and in studies measuring its protein expression (24, 26). Evidence also points to the involvement of IL-12, with significantly higher levels observed in suicidal patients with MDD than in their non-suicidal counterparts (26). Observations by multiple research groups have also indicated a correlation between IFN-γ and increased suicide risk (11), further underscoring the activation of a broad pro-inflammatory network. In our experimental study, we also observed elevated levels of pro-inflammatory factors IL-12 and IFN-γ in patients with high suicide risk. Although there was no statistically significant difference in IL-6, a certain trend for a difference was observed (P = 0.0633). This may be due to the limited sample size, which may have resulted in insufficient statistical efficacy. Future studies with larger sample sizes are warranted.

Concurrent with pro-inflammatory activation, suicide risk is associated with a marked deficit in immunoregulatory mechanisms. IL-4 has emerged as a key protective factor, with lower levels characterizing individuals who attempt suicide (27) and contributing significantly to the variance in suicidal behaviors (28), while the IL-2/sIL-2R pathway shows significant downregulation (21, 28). Furthermore, IL-10 has demonstrated an inverse correlation with suicidality (29). In contrast, other studies have found that significantly higher plasma IL-10 levels were observed in the suicidal group (30). Similarly, we observed elevated IL-4 levels in the high-risk group for suicide. In summary, compiled evidence suggests that suicidality is associated with a distinct neuroimmune signature, primarily defined by the co-occurrence of pro-inflammatory pathway activation and a breakdown in immunoregulatory homeostasis.

In the preliminary two-sample MR study analyzing 16 immune factors, IL-27 demonstrated a positive correlation with suicidal ideation, whereas TRAIL showed a negative correlation. Complementing the MR findings, our observational data further revealed a distinct inflammatory signature for high-risk individuals, characterized by elevated peripheral blood levels of IL-4, IL-12, IFN-γ, and, notably, IL-27.

Our MR analysis identifies a genetic association between IL-27 and suicidal ideation, which is biologically corroborated by elevated peripheral IL-27 levels in high-risk suicidal individuals, highlighting its potential as a clinically viable biomarker for assessing suicidal risk in depressive patients.

The cytokine IL-27, classified within this family, has been found to support both proinflammatory and anti-inflammatory effects. Th1 cell formation is induced by IL-27 in the context of bacterial or parasitic infections (31). Moreover, it promotes the differentiation of T follicular helper cells by inducing IL-21 production, thereby modulating B cell activity (32). The immunoregulatory roles of IL-27 encompass the suppression of Th2 and Th17 cell differentiation, along with the promotion of IL-10-secreting Type 1 regulatory T (Tr1) cells, which serve to mitigate immunopathological responses during infections (33).

IL-27 also regulates inflammatory processes within the central nervous system. Investigators examined the expression patterns of IL-27 subunits in brain specimens from patients diagnosed with multiple sclerosis (MS) and compared them with control samples from neurologically healthy donors without any clinical or neuropathological signs of central nervous system disorders (34).

Immunohistochemical analyses revealed that IL-27 subunits were predominantly localized to astrocytes, microglia, and macrophages within the central nervous system. Notably, MS patients exhibited significantly elevated expression levels of these subunits compared to healthy controls. Furthermore, consistent with the lymph node expression profile, the brain demonstrated a partial co-expression pattern of IL-27 subunits (35).

The dysregulation of neuroimmune pathways may represent a potential mechanism by which IL-27 promotes suicide risk. IL-27 exemplifies a quintessential immunomodulatory cytokine with a profoundly dual role in CNS pathology. This cytokine is secreted by and interacts with a diverse network of neural and immune cell populations, including infiltrating microglia, macrophages, astrocytes, and even neurons. Thus, it is a central mediator of the neuroimmune axis (36, 37). Under many experimental conditions, IL-27 acts as a potent neuroprotective agent. It promotes neuronal survival and tissue integrity through a multi-faceted mechanism that includes the modulation of pro- and anti-inflammatory cytokine networks (notably, IL-10 induction), regulation of neuroinflammatory signaling cascades (e.g., JAK-STAT, NF-κB) (38, 39), attenuation of oxidative stress responses, suppression of apoptotic pathways, enhancement of cellular autophagy, and epigenetic regulation (40). This protective role is well documented in models such as those of experimental autoimmune encephalomyelitis (EAE), cerebral hemorrhage, and retinal degeneration, where its effects are often mediated through the suppression of pathogenic T helper cells and a direct anti-inflammatory action on microglia (41). Nevertheless, this ostensibly beneficial profile is counterbalanced by a paradoxical capacity for harm under specific pathological conditions. IL-27 can exert pro-inflammatory and cytotoxic effects, triggering inflammatory responses in certain macrophage populations and promoting apoptosis. This function, while beneficial for anti-tumor immunity, may exacerbate neurodegeneration. The ultimate impact of IL-27 appears to be a function of the specific cellular targets, disease microenvironment, and temporal stage of the pathology, with evidence suggesting it may play a more pro-inflammatory role early in disease courses (42). This profound duality underscores the fact that IL-27 is neither exclusively neuroprotective nor neurotoxic; rather, it is a pivotal immunomodulator, the net effect of which is determined by the delicate balance of signaling pathways it engages within a given context.

The observed association between IL-27 and suicidal ideation in the MR analysis suggests that genetic variants influencing IL-27 levels may exert long-term effects on the neuroimmune pathways. IL-27 can modulate immune responses in the central nervous system, potentially affecting neuroinflammation, neuronal survival, and neurotransmitter systems. IL-27 may serve as a potential biomarker for assessing suicidal ideation. However, considering its dual immunomodulatory role in neuroimmunity, further investigation into its upstream and downstream signaling pathways, aiming to selectively amplify its protective functions while mitigating its detrimental potential, is necessary before it can be targeted for therapeutic intervention.

Beyond the central role of IL-27, our clinical data revealed a broader inflammatory signature associated with high suicide risk, characterized by co-elevated levels of IL-4, IL-12, and IFN-γ. It is crucial to note that these cytokines did not show significant causal associations in our MR analysis. This pattern suggests that IL-12 (a driver of Th1 responses) and IFN-γ (its key effector) may represent downstream effectors or correlative markers of the acutely dysregulated immune state, rather than upstream causal drivers, in severely suicidal individuals. The simultaneous elevation of IL-4, a Th2 cytokine, hints at a complex, dysregulated immune response that may involve failed compensatory mechanisms. Collectively, IL-27, IL-12, IFN-γ, and IL-4 may form a distinct inflammatory profile that characterizes patients with MDD who are at the highest risk for suicide.

In contrast to the pro-inflammatory profile, our MR analysis identified TRAIL as a factor with a significant protective effect against suicidal ideation. This suggests that genetically determined higher TRAIL levels may confer resilience. TRAIL plays a regulatory role in immune-mediated neuroinflammation that intersects with IL-12 signaling (43). While IL-12 primarily drives pro-inflammatory responses, TRAIL exhibits dual functionality. It can induce apoptosis in activated immune cells, thereby limiting excessive inflammation. However, it may also contribute to neuronal cell death under certain pathological conditions (44).The inverse association between TRAIL and suicidal ideation observed in the MR analysis suggests that TRAIL may play a neuroprotective role. Circulating TRAIL levels inversely correlate with chronic inflammatory states, with lower levels observed in individuals experiencing cytokine-driven hyperinflammation (44).

### 4.1 Limitations and future directions

This study has several limitations that warrant consideration. First, the modest size of our clinical cohort may limit the generalizability of the findings. Second, the MR analyses relied on publicly available summary-level GWAS, which may introduce population- or platform-specific biases and constrained harmonization to the released data. Importantly, the scope of the genetic outcome warrants clarification: UK Biobank Field 20513 (“recent thoughts of suicide or self-harm,” PHQ-9 item 9) conflates suicidal ideation with non-suicidal self-harm and is not restricted to individuals with MDD. Third, although blood samples were collected before the initiation of new treatment regimens, detailed medication histories were not available for rigorous statistical adjustment of potential confounding effects of existing medications. Furthermore, we did not measure TRAIL levels in our clinical cohort, leaving its physiological role in this context to be empirically validated.

Future studies should aim to include larger, more diverse populations to validate these findings. Moreover, they should incorporate strictly controlled, prospectively collected medication data to better account for confounding effects of medications. Furthermore, subsequent investigations should continue to measure IL-27 levels in clinical cohorts to evaluate its predictive utility, elucidate the molecular and cellular mechanisms through which IL-27 influences suicidal ideation, and examine how environmental factors modulate IL-27 levels and their relationship with suicide risk.

## 5 Conclusion

This study highlights the complex roles of cytokines in depression and suicidal ideation. IL-27 emerged as a promising peripheral biomarker showing a potential relationship with high suicidal ideation, suggesting a genetic influence on neuroimmune processes. Future research should aim to systematically validate these observations in large-scale clinical populations and explore immune mechanism and cytokine-based therapeutic strategies to mitigate suicidal ideation.

## Data Availability

The datasets generated and/or analyzed during the current study are available in the Zenodo repository, https://zenodo.org/records/17362584.
